# The informed consent in Southern Italy does not adequately inform parents about infant vaccination

**DOI:** 10.1186/1471-2458-14-211

**Published:** 2014-02-28

**Authors:** Francesco Attena, Amanda Valdes Abuadili, Sara Marino

**Affiliations:** 1 Department of Experimental Medicine, Second University of Naples, via Luciano Armanni, 5, 80138 Naples, Italy; 2 School of Hygiene and Preventive Medicine, Second University of Naples, via Luciano Armanni 5, Naples, Italy

**Keywords:** Information, Informed consent, Infant vaccination

## Abstract

**Background:**

Vaccination centres in the Campania Region, southern Italy, vaccinate children with a hexavalent vaccine that contains the mandatory vaccines diphtheria, tetanus, poliomyelitis, and viral Hepatitis B. This vaccine also includes two non-mandatory vaccines, pertussis and Haemophilus influenzae type B. Information about these optional vaccines should be communicated to the parents, and informed consent should be obtained from parents before vaccination. We explored whether informed consent was delivered to the parents, whether they signed the consent form, and whether they read and acquired the information about the vaccination that their child would receive.

**Methods:**

Childhood immunisations are provided at specific public health vaccination centres, "Unità Operative Materno-infantili’s" (UOMIs). We selected four UOMI from the Campania Region where we interviewed 1039 parents bringing their children for the 1st, 2nd, or 3rd doses of hexavalent vaccine. The consent forms were collected from the four vaccination centres and were analysed with respect to clarity and completeness.

**Results:**

Most of the respondents (89.5%) were mothers between 20 and 39 years of age (80.4% vs 59.6% of the fathers), they were married (87.2% vs 93.5% of the fathers), and only one-half of them were employed (50.2% vs 92.6% of the fathers). The informed consent form was received from 58.1% of the parents and signed by 52.8%, but read by 35.0% of them. Only 1.5% of parents knew which vaccines were mandatory, and 25.0% of them believed that the entire hexavalent vaccine was mandatory. When we asked the parents which non-mandatory vaccinations were administered to their children, only 0.5% indicated the Haemophilus influenzae type B and none indicated the pertussis vaccine. Thirty-six per cent of the parents replied that their child had not received any non-mandatory vaccines. No parents were informed by the operators that their children would receive non-mandatory vaccines.

**Conclusion:**

In our study, consent procedures did not allow parents to acquire correct information about vaccine options for their children. Furthermore, not one health care provider informed parents that their child was receiving non-mandatory vaccines. The informed consent process and the individual health care providers did not properly inform parents about the vaccines administered to their children.

## Background

Mandatory vaccination against diphtheria, tetanus, poliomyelitis, and viral Hepatitis B was instituted in Italy during the past century. As new and more effective and safe vaccines have been developed, Italian health authorities have decided that some of these new vaccines are not mandatory, but are recommended or "strongly recommended". In recent years, health authorities have urged vaccination centres to use a hexavalent vaccine for infant immunisation that includes pertussis and Haemophilus influenzae type B, in addition to the four mandatory vaccines. Information about these optional vaccines should be communicated to the parents, and informed consent should be obtained from parents before vaccination. However, in Italy the differences between mandatory and recommended vaccinations are weaker than in the past, and the concept of a "mandatory" vaccine is now ambiguous. Before 2008, children were not allowed to attend school if they were not vaccinated, but this rule has now been abrogated. Administrative sanctions for an unvaccinated child are rarely applied. If a parent refuses to vaccinate his/her child, the parent will receive an informative talk at the local health authority in an attempt to gain compliance [1]. Legislatively, each Italian region is relatively autonomous, so the laws regarding vaccination vary throughout the country (e.g., in the Veneto Region, laws mandate that vaccinations are not mandatory).

The issue of conveying correct information to the patient population has been well-investigated, as has the safety and effectiveness of, and contraindications for, infant vaccines. Moreover, the era of patient-centred health care has resulted in improvements in patient empowerment, health literacy [2], evidence-based patient information [3], and risk communication [4]. We explored whether informed consent was delivered to the parents, whether they signed the consent form, and whether they read and acquired the information about the vaccination that their children would receive. This issue is relevant because informed consent plays an important role in parents’ knowledge about infant vaccination policies, and because parents are the main decision-makers with respect to whether certain vaccinations are given to their children. Moreover, in general, most options available for medical interventions are very complex. It is difficult to explain the details of the therapeutic alternatives, the potential outcomes, and the balance between risk and benefits. However, for infant vaccinations, the information provided by informed consent is easy to explain and to understand.

## Methods

### Participants and setting

In the Campania Region of southern Italy, vaccine type decisions, purchasing, and supply management is centralised at the Local Health Authority (ASL) and regional level. Childhood immunisations are provided at specific public health vaccination centres, "Unità Operative Materno-infantili’s" (UOMIs). We selected one vaccination centre from each of the four cities with the largest populations in the Campania Region (Napoli, Salerno, Caserta, and Avellino). We randomly selected one of the ten vaccination centres that are present in the city of Napoli. Only one vaccination centre is present in the cities of Salerno, Caserta, and Avellino. The policy at each centre was to provide informed consent about vaccination as a written document that the parents were to be instructed to read and sign.

At each centre, two expert health care operators interviewed all parents bringing their children for the 1st, 2nd, or 3rd doses of hexavalent vaccine. In presence of both parents, the operators interviewed only the mothers. A written informed consent for the study was obtained from each participant. The interviews were carried out immediately after vaccination, and were conducted one or two days each week between January and April, 2013, during all the hours that the centres were open to the public.

### Sample size

The sample size of approximately 1000 subjects was obtained by assuming a 95% prevalence of the main outcome, a precision of ±1.2%, a confidence level of 95% and a power of 80%. A total of 1039 questionnaires were completed (Napoli: 329; Avellino: 254; Salerno: 251; Caserta: 205). Using a univariate analysis and chi-square test, we analysed the parents’ responses according to demographic characteristics and to vaccination centre. A value of *p* < 0.05 was considered to be statistically significant.

### Questionnaire

The following questions were used to investigate the parents’ knowledge and the vaccine information that they were provided: "Did you receive a written informed consent before vaccination?" (yes/no) (main outcome); "Did you sign the informed consent" (yes/no/I don’t remember); "Did you read the informed consent?" (yes/no/partially); "Have your children received other vaccinations in addition to the mandatory ones?" (yes/no); "if yes, which one?" (open); "Did anyone ask you for consent to give your child the two non-mandatory vaccinations that are included in the hexavalent vaccine?" (yes/no); "Do you know which vaccinations are mandatory?" (open/I don’t know). The answers to this question were coded as: "correct" when only the four mandatory vaccinations were indicated, and "not correct" for all other responses, except that the response "hexavalent" (also not correct) was calculated separately. Data on the sociodemographic characteristics age, sex, marital status, education, and occupation were also collected. The Ethics Committee of the Second University of Naples approved this study (reference number 41/2012).

### Informed consent forms

The infant vaccination informed consent forms that were presented by the health operators at the four vaccination centres were analysed with respect to completeness and clarity. Completeness was evaluated according to a definition that conformed with government recommendations: "Informed consent is a procedure to ensure that a patient, client, and research participants are aware of all the potential risks and costs involved in a treatment or procedure. The elements of informed consent include informing the client of the nature of the treatment, possible alternative treatments, and the potential risks and benefits of the treatment".

Clarity was evaluated by the achievement of an agreement between all of the authors. However, all informed consent forms were also previously validated for clarity and completeness, by the ethics committees of the respective local health authorities.

## Results

### Informed consent

The informed consent forms from the four centres were similar and were sufficiently clear and complete with respect to the aims of the questionnaire. They included information about which vaccines are mandatory, which are recommended, which vaccines were to be administered, the content of the hexavalent vaccine, and information about safety, effectiveness, contraindications, and how to respond to an adverse reaction.

### Questionnaire

All children at each of the four centres received the hexavalent vaccine; 1039 parents completed the questionnaire and 34 refused to answer. Most of the respondents (89.5%) were mothers. Most of the mothers were between 20 and 39 years of age (80.4% vs 59.6% of the fathers) and were married (87.2% vs 93.5% of the fathers); only one-half of them were employed (50.2% vs 92.6% of the fathers) (Table 1). The informed consent form was received from 58.1% of the respondents. At one centre, none of the parents received the consent form, while at the other three centres 93.7%, 71.6%, and 70.0% of the parents received the form (data not reported in table). Among those who received an informed consent, it was signed by 90.9% (9.1% did not remember to sign it), while in total it was signed by 52.8% of the parents. Only 66.3% of the parents who signed the consent form read the document, while overall only 35.0% of the parents read the informed consent form (Table 2).

**Table 1:** Selected characteristics of the study population

***Characteristic***	***Mother***		***Father***	
	**N**	**(%)**	**N**	**(%)**
*Age*				
15-19	12	(1.3)	-	
20-29	229	(24.7)	15	(13.7)
30-39	516	(55.7)	50	(45.9)
40-49	165	(17.8)	39	(35.8)
50-59	5	(0.5)	5	(4.6)
*Missing*	3		0	
*Marital status*				
Married	807	(87.2)	101	(93.5)
Other	118	(12.8)	7	(6.5)
*missing*	5		1	
*Education*				
Primary school	30	(3.2)	2	(1.8)
Middle school	215	(23.2)	18	(16.5)
High school	370	(39.9)	38	(34.8)
College degree	312	(33.7)	51	(46.9)
*Missing*	3		0	
*Occupation*				
Employed	465	(50.2)	100	(92.6)
Unemployment	461	(49.8)	8	(7.4)
*Missing*	4		1	
Total	930	(89.2)	109	(10.5)

**Table 2:** Parents who received, signed, and read the informed consent form before their children were vaccinated

	**Received**	**Signed (among who received)**	**Read (among who signed)**
Yes	604	549	364
	58.1%	90.9%	66.3%
No	435	0	130
	41.9%	0%	23.7%
Partly	-	0	48
		0%	8.7%
Don’t remember	-	55	7
		9.1%	1.3%
Total	1039	604	549
	100%	100%	100%
Total who signed		549/1039	-
		52.8%	
Total who read		-	364/1039
			35.0%

Only a few of the parents (1.5%) knew which vaccinations were mandatory; 25.0% of them believed that the entire hexavalent vaccine was mandatory. When we asked the parents which non-mandatory vaccinations were administered to their children, only 0.5% indicated the Haemophilus influenzae type B vaccine. None of the parents indicated that the pertussis vaccine had been given to their child. Thirty-six per cent of the parents replied that their child had not received any non-mandatory vaccines. No parents were informed by the operators that their children would receive non-mandatory vaccines (Table 3).

**Table 3:** Knowledge, information, and consent about mandatory and recommended vaccinations

	**Not correct**	**Hexavalent**		**Correct**	**Don’t know**	**Total**
Do you know which vaccines are mandatory?	370	260		16	393	1039
	35.7%	25.0%		1.5%	37.8%	100%
	**Don’t know**	**No**	**Other**	**Hib**	**Pertussis**	**Total**
Did your children receive any non-mandatory vaccines?	83	374	577	5	0	1039
	8.0%	36.0%	55.5%	0.5%	0%	100%
	**Yes**			**No**		**Total**
Did anyone ask you for consent to give your child the two non-mandatory vaccinations that are included in the hexavalent vaccine?	0			1039		1039
	0%			100%		100%

With the exception of the difference between centres’ distribution of informed consent forms, there were no significant differences when the data were disaggregated according to sociodemographic characteristics, vaccination centres, and signature or reading of the informed consent form.

## Discussion

Surprisingly, and despite three potential sources of information—basic level (e.g., newspaper, internet), written informed consent, and communication from health professionals—almost all of the parents did not know which vaccinations were mandatory and which vaccines were administered to their children.

Health information from the media is not always correct, complete, and transparent [5,6]; the media is, however, considered to be an important source of this information. According to our results, the media was not sufficient to provide correct information to our sample about infant vaccination.

Problems and limitations of informed consent have been evaluated in different health care settings (i.e., clinical, surgery, emergency, and research) [7-12] according to the four principles of biomedical ethics [13] considered in the relationship between health care services and the individual patient, i.e. autonomy, beneficence, nonmaleficence, and justice. Individual patient autonomy is important in the public health care setting, as it is in the clinical setting. However, in the public health setting, beneficence and nonmaleficence have both individual and collective consequences, so individual consent alone is inadequate for the development of public health policies [14,15]. Vaccination is unique, because compared with other public health interventions, vaccination has greater individual than collective implications. The principles of beneficence and nonmaleficence have deeper individual relevance. Informed consent for vaccination, therefore, can have a greater impact than other general public health issues.

Among public health experts and ethicists, the role of informed consent in vaccination and in infant vaccination has been debated [16,17]. Those who argue the importance of the signed consent emphasise, in analogy to all other health interventions, the principles of autonomy and freedom of choice [18-20]. Other authors disapprove of the use of informed consent because they consider the collective benefits of mandatory vaccination to be more important [21]; because informed consent can lower the vaccination coverage [22]; because vaccination is a low-risk and high-benefit preventative measure, and should be excluded from informed consent, similar to other low-risk and high-benefit interventions (e.g. antibiotic treatment) [23].

In our study, only one-half of the parents received and signed the consent, and results varied between the four centres from 0% to 93.7%. Moreover, only 35.0% of the parents read the informed consent form, and none of them were able to report on its contents. Perhaps they were not interested in the information, or perhaps they thought that consent was irrelevant and considered vaccination to be mandatory and unavoidable. Also who stated to have read it, may have signed the document without reading it, or did not read it completely. Health care professionals may consider themselves exempt from providing information to parents because they view this to be the role of written informed consent. Thus, the results of this study indicate that consent procedures, and in particular written informed consent, did not allow parents to acquire correct information about vaccine options for their children.

These data show that in our area, informed consent as a process and as a written document that requires a signature is not sufficiently implemented at the vaccination centres. This is in spite of the implementation of a European Directive in December 2009 that was approved in Italy, which made informed consent mandatory for all types of vaccination, for all citizens. Since each vaccination centre depends on the local health authority, the latter is responsible for this deficiency. These data also suggest that training and information programs for both citizens and health personnel should be implemented to facilitate a more appropriate application of the whole process of informed consent.

## Conclusions

In our study, neither written informed consent nor health care providers informed parents about the vaccines administered to their children and that their child was receiving non-mandatory vaccines. Therefore, in this particular health setting, consent procedures, and in particular written informed consent, did not allow parents to acquire correct information about vaccine options for their children. External validity is the main limitation of this study, so it should be repeated elsewhere in Italy or in other countries.

## Competing interests

All authors declare that they have no competing interests.

## Authors’ contributions

AF: conception and design of the study, interpretation of data, drafting the article. MS: acquisition and analysis of data. AVA: acquisition and analysis of data. All authors read and approved the final manuscript.

## Pre-publication history

The pre-publication history for this paper can be accessed here:

http://www.biomedcentral.com/1471-2458/14/211/prepub
